# Assessment of Kidney Function in Mouse Models of Glomerular Disease

**DOI:** 10.3791/57764

**Published:** 2018-06-30

**Authors:** Megan Stevens, Sebastian Oltean

**Affiliations:** ^1^Institute of Biomedical and Clinical Sciences, Medical School, University of Exeter; ^2^School of Physiology, Pharmacology and Neurosciences, University of Bristol; ^3^Bristol Renal, School of Clinical Sciences, University of Bristol

**Keywords:** Medicine, Issue 136, Glomerular phenotype, mouse models of renal disease, glomerular permeability, glomerular ultra-structure, mechanisms of glomerular function, VEGF-A

## Abstract

The use of murine models to mimic human kidney disease is becoming increasingly common. Our research is focused on the assessment of glomerular function in diabetic nephropathy and podocyte-specific VEGF-A knock-out mice; therefore, this protocol describes the full kidney work-up used in our lab to assess these mouse models of glomerular disease, enabling a vast amount of information regarding kidney and glomerular function to be obtained from a single mouse. In comparison to alternative methods presented in the literature to assess glomerular function, the use of the method outlined in this paper enables the glomerular phenotype to be fully evaluated from multiple aspects. By using this method, the researcher can determine the kidney phenotype of the model and assess the mechanism as to why the phenotype develops. This vital information on the mechanism of disease is required when examining potential therapeutic avenues in these models. The methods allow for detailed functional assessment of the glomerular filtration barrier through measurement of the urinary albumin creatinine ratio and individual glomerular water permeability, as well as both structural and ultra-structural examination using the Periodic Acid Schiff stain and electron microscopy. Furthermore, analysis of the genes dysregulated at the mRNA and protein level enables mechanistic analysis of glomerular function. This protocol outlines the generic but adaptable methods that can be applied to all mouse models of glomerular disease.

**Figure:**
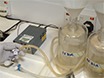


## Introduction

The use of murine models to mimic human kidney disease is becoming increasingly common. Such murine models include spontaneous models such as spontaneously hypertensive rats (SHR)^1^, streptozotocin (STZ)-induced diabetic rats and mice^2^, and the db/db type II diabetic mice^3^, genetically engineered models such as primary podocyte-specific focal segmental glomerular sclerosis (FSGS) models^4^, the podocyte-specific vascular endothelial growth factor A (VEGF-A) knock-out (VEGF-A KO) model^5^, and Alport syndrome models^6^, and acquired models such as the 5/6 nephrectomy^7^ and the unilateral ureteral obstruction (UUO) model^8^. In order to assess the different aspects of glomerular function in these models, several techniques are available. The purpose of this method paper is to demonstrate a comprehensive work-up that should be performed in mouse models of kidney disease in order to fully assess glomerular function.

The rationale behind the use of this method is that it enables the glomerular phenotype to be fully evaluated from multiple aspects. This includes assessing the glomerular permeability, both to protein and to water, the glomerular structural abnormalities, and changes in the expression/splicing of mRNAs and proteins essential for normal glomerular function. By using this method, the researcher is able to determine the kidney phenotype of the model and assess the mechanism as to why the phenotype develops. This is vital information on the mechanism of disease, which is required when examining potential therapeutic avenues in these models.

In the literature, it is a common occurrence to be presented with a mouse model of glomerular disease where the phenotype is determined by an increased level of albumin in the urine. However, there is evidence to suggest that a single method to determine glomerular function is not always effective; measuring the urinary albumin excretion rate or the urinary albumin creatinine ratio (uACR) only provides information on total renal function, and not of the individual glomeruli. Previous studies have demonstrated that the permeability can vary in different glomeruli from the same kidney^5^,^9^,^10^. In addition, assessment of the permeability of individual glomeruli is a more sensitive way of assessing glomerular function; the technique of measuring the individual glomerular water permeability (L_p_A/V_i_) has shown to be more sensitive to changes in glomerular function than measuring the uACR^9^. This assay is beneficial in mouse models that are resistant to proteinuria, such as those on a c57BL/6 background^11^. The advantage of the present method paper is that it examines both the total renal permeability to albumin as well as the individual glomerular permeability to water.

Examination of glomerular structural abnormalities is often assessed by a battery of stains such as Periodic Acid Schiff (PAS), trichrome, and silver stains. These enable a trained renal pathologist to evaluate the level of renal disease via a scoring method. Although all good methods, changes to the glomerular macro-structure are not always observed in acute kidney injury models^12^. This method proposes that in addition to carrying out the renal histology techniques described above, the glomerular ultra-structure should also be assessed via electron microscopy (EM). A stained glomerulus can look relatively normal under a regular light microscope; however, upon assessment with EM, small changes in the glomerular basement membrane (GBM) width, podocyte foot process effacement, endothelial fenestrations, and the sub-podocyte space coverage is analyzed. Therefore, it is vital that both the glomerular ultra-structure and micro-structure is assessed to determine the mechanism of glomerular dysfunction.

In addition to assessing glomerular structural abnormalities, changes in mRNA and protein expression and splicing, as well as protein activation (*e.g.*, phosphorylation), should be examined to further elucidate the mechanisms of glomerular disease. When looking at glomerular disease, or, for example, when KO/over-expressing a gene specifically in glomerular cells, such as in the podocyte-specific VEGF-A KO mouse^5^, it is important that the protein and mRNA changes are examined only within the glomerular cells, and not the whole kidney. This protocol describes a method in which the glomeruli are isolated from the mouse kidney cortex, and then the protein/RNA are isolated. This allows specific analysis of the protein/mRNA dysregulation in the glomeruli of the disease model.

This protocol describes a full kidney work-up that should be carried out in mouse models of glomerular disease, enabling a vast amount of information regarding kidney and glomerular function to be obtained from a single mouse. The methods allow for detailed functional, structural, and mechanistic analysis of glomerular function, which can be applied to all mouse models of glomerular disease.

## Protocol

All experiments were conducted in accordance with UK legislation and local ethical committee approval. Animal studies were approved by University of Bristol Research Ethics Committee.

### 1. Urinary Albumin Creatinine Ratio (uACR)

NOTE: The uACR is used to assess the permeability of the GFB to albumin. The presence of albumin in the urine indicates increased permeability across the GFB, which is normalized to creatinine to control for variations in urine flow rate. Albuminuria is a common marker for chronic kidney disease.

1. Collect urine at the experimental baseline and at regular intervals (once weekly to once monthly) up until the experimental endpoint.
2. Set up mouse metabolic cages with water and enrichment diet. Place mice (male, aged 6 - 8 weeks) in individual cages for 6 h in a quiet room.
3. Return mice to regular housing and collect urine from the empty cages. A minimum of 50 µL is required. NOTE: If the mouse does not produce urine in the given time, repeat on another day in a warmer room.
4. Centrifuge the urine at 500 x g for 10 min. Collect the urine and retain sediment to assess podocyte loss. Store the urine at -20 °C in the short-term at this point.
5. Dilute the urine into 1% bovine serum albumin (BSA) in 1x Tris buffered saline (TBS pH 7.5) at a 1:500 to 1:10000 dilution, depending on the severity of the albuminuria. NOTE: The end volume should be >400 µL. Optimize to determine the right dilution at each time point.
6. Quantify the urinary albumin concentration using a mouse albumin enzyme linked immunosorbent assay (ELISA) per the manufacturer's instructions.
  1. Briefly, coat the ELISA plate, which is optimized for protein binding, with 100 µL of the anti-mouse albumin primary antibody (10 µg/mL in 0.5 M carbonate-bicarbonate pH 9.6) for 1 h at room temperature.
  2. Wash excess antibody from the wells five times with 1x TBS plus 0.05% Tween (pH 8.0), and add 200 µL of blocking solution (1x TBS with 1% BSA pH 8.0) overnight at room temperature.
7. Wash plate five times and add 100 µL of the standards (serial dilution: 2000 µg/mL to 15.63 µg/mL in 1x TBS with 1% BSA, pH 8.0), the blank, and the diluted samples in triplicate. Leave for 1 h at room temperature then wash plate five times.
  1. Add 100 µL of HRP detection antibody to each well (10 ng/mL in 1xTBS with 1% BSA, pH 8.0) and incubate at room temperature for 1 h.
  2. Wash the plate five times and add 100 µL of enzyme substrate solution to each well. Leave the plate in the dark to develop for 15 min and stop the reaction by adding 100 µL of 0.18 M sulfuric acid (H_2_SO_4_). CAUTION: H_2_SO_4_ is corrosive.
8. Determine the albumin concentration of each sample by reading the plate at an absorbance of 450 nm. Use the standard curve to quantify the albumin present in each sample. If the technical repeats have a CV value greater than 5%, repeat the assay for those samples.
9. Alternatively, assess the urinary albumin concentration using electrophoresis. Add 5 µL of 4x protein sample loading buffer to 15 µL of urine. Heat samples to 95 °C for 10 min and then load into a 4 - 12% Tris precast gel.
  1. Run the gel at 100 V and stain overnight in Coomassie blue per manufacturer's protocol.
  2. After imaging the gel, use densitometry to assess fold change albumin concentration relative to control. NOTE: If no bands are observed for albumin when expected, assess the protein concentration of the urine and adjust the volume of urine added to the gel.
10. Dilute the raw urine sample in dH_2_O at 1:1, 1:5, and 1:10. NOTE: The end volume should be >70 µL. Optimize to determine the right dilution of each sample.
11. Quantify the urinary creatinine concentration using a chemical assay per the kit instructions. Briefly, load 20 µL of creatinine standards, blank, or diluted urine to a 96 well plate in triplicate.
12. Determine the creatinine concentration of each sample by reading the plate at an absorbance of 490 nm before and after the addition of the acid solution (CAUTION, corrosive; avoid contact with skin). NOTE: The difference between the absorbance values is directly proportional to the creatinine concentration in each sample. A standard curve is generation from the standards. If the technical repeats have a CV value greater than 5%, repeat the assay for those samples.
13. Generate the uACR (µg/mg). Normalize the data to the baseline value of each mouse for graphical representation.

### 2. Tissue and Blood Collection

NOTE: The kidney and glomerular tissue can be used to assess structural, protein, and mRNA expression markers of renal disease. The blood can be used to assess markers of renal function, such as creatinine, which can be up-regulated in renal disease, indicating a reduction in the filtration capacity of the glomeruli.

1. Prepare the following solutions: fresh 2.5% glutaraldehyde in 0.1 M sodium cacodylate (pH 7.3), 4% paraformaldehyde in 1x phosphate buffered saline (PBS), mammalian Ringer's solution (115 mM sodium chloride (NaCl), 10 mM sodium acetate (CH_3_COONa), 1.2 mM sodium phosphate (Na_2_HPO_4_), 25 MM sodium bicarbonate (NaHCO_3_), 1.2 mM magnesium sulfate (MgSO_4_), 1 mM calcium chloride (CaCl_2_), 5.5 mM D(+)glucose, pH 7.4) with 1% BSA, and 1x PBS. CAUTION: 2.5% glutaraldehyde: toxic, sensitizer, irritant; use in a fume cabinet. 0.1 M sodium cacodylate: toxic, use in a fume cabinet. 4% PFA: fixative, use in fume cabinet
2. Prepare the following materials: isoflurane, small ethylenediaminetetraacetic acid (EDTA)-coated blood tubes, 23 - 25G needles, 5 mL EDTA coated syringes, 10 mL glass vials, 10 mL plastic vials, 0.5 mL plastic tubes, disposable tissue molds, dry ice, liquid N_2_, mouse surgical tools, and optimal cutting medium (OCT).
3. Place the mouse under deep anesthesia, which is verified by the mouse being non-responsive to a needle prick to the foot pad, using an isoflurane chamber, or equivalent routes of terminal anesthesia such as injectable anesthetic agents (pentobarbital, 50 mg/kg intraperitoneal [IP]; avertin, 240 mg/kg IP), or carbon dioxide (CO_2_) exposure (75% CO_2_/25% O_2_).
4. Cull mouse via cardiac puncture into the left ventricle and collect as much blood as possible. Transfer to the EDTA-coated blood tube for up to 4 h. If preferred, mice can be culled via cervical dislocation with care taken not to rupture the jugular vein.
5. Dissect out the kidneys through the abdomen and wash in ice cold 1x PBS.
6. To examine the cortical glomeruli, remove one pole of kidney cortex and cut into 1 mm^3^ pieces. To examine the deep juxta-medullary glomeruli, repeat the same technique with tissue from the medulla. Place in 5 mL of 2.5% glutaraldehyde solution in a glass EM vial. Store at 4 °C. CAUTION: 2.5% glutaraldehyde solution: toxic, sensitizer, irritant; use in a fume cabinet NOTE: process within 1 month for best results.
7. For histology, remove the upper third of a kidney, to ensure both cortical and juxta-medullary glomeruli will be present, and fix in 5 mL of 4% paraformaldehyde at 4 °C for 24 h. Transfer to 5 mL of 70% EtOH for 24 hours before embedding in paraffin. CAUTION: 4% PFA: fixative, use in fume cabinet
8. For immunofluorescence, place a third of the kidney, to ensure both cortical and medullary glomeruli will be present, into the tissue mold and coat in OCT. Place on dry ice to freeze and store at -80 °C.
9. For protein and RNA, place 3 x 2 mm^3^ pieces of kidney cortex into 0.5 mL plastic tubes and snap freeze in liquid N_2_. Store at -80 °C. For long-term tissue storage for RNA, place tissue in 5 volumes of RNA stabilization solution and store at -80 °C.
10. For isolation of glomeruli, slice up the remaining kidney tissue and place in 5 mL of mammalian Ringer's solution with 1% BSA on ice. Prepare to sieve glomeruli immediately.

### 3. Plasma Creatinine

NOTE: Plasma creatinine can be up-regulated in renal disease, indicating a reduction in the filtration capacity of the glomeruli. The blood urea nitrogen (BUN) levels can also be assessed, although the protocol is not described here.

1. Centrifuge the blood sample at 500 x g for 15 min at 4 °C.
2. Collect the plasma, which can be stored at -20 °C in the short-term at this point.
3. Quantify the plasma creatinine concentration using a chemical creatinine assay per the instructions above for urinary creatinine in protocol 1.11.
4. Determine the creatinine concentration of each sample by reading the plate at an absorbance of 490 nm before and after the addition of the acid solution. NOTE: The difference between these absorbance values is directly proportional to the creatinine concentration in each sample. A standard curve is generation from the standards. If the technical repeats have a CV value greater than 5%, repeat the assay for those samples.

### 4. Isolation of Glomeruli

NOTE: Glomeruli can be isolated to assess the permeability of individual glomeruli *ex vivo*, as well as the expression of specific protein and mRNA markers of glomerular disease.

1. Take the kidney tissue placed in mammalian Ringer's solution with 1% BSA and dissect the glomeruli using a standard sieving technique^13^. Briefly, stack the 70 µm (bottom), 100 µm, 125 µm, 175 µm, 250 µm, and 425 µm (top) sieves on to a glass beaker.
2. Mash the kidney, using a syringe plunger, into the 425 µm sieve and push through using ice cold mammalian Ringer's solution with 1% BSA. As the bits of kidney are pushed through, remove the top sieve and proceed to do the same on the next. Repeat until only the 100 µm and 70 µm sieves remain.
3. Transfer the glomerular harvest retained by the 100 µm and 70 µm sieves to 10 mL of fresh mammalian Ringer's solution with 1% BSA, on ice. NOTE: If the number of glomeruli per mL Ringer's solution is few, reduce the volume of Ringer's solution used to collect the glomeruli from the last two sieves.
4. Remove 5 mL of the solution containing glomeruli into two separate tubes (2.5 mL each) and centrifuge at 1,000 x g for 10 min at 4 °C. Remove the supernatant and snap freeze the glomeruli in liquid N_2_ before storing at -80 °C for protein and RNA extraction at a later date.
5. Place the remaining solution containing glomeruli in a water bath at 37 °C for measurement of the glomerular L_p_A/V_i_ *ex vivo.* Complete within 3 h of removing the kidney.

### 5. Glomerular Water Permeability (L_p_A/V_i_)

NOTE: The glomerular L_p_A/V_i_ assay enables the *ex vivo* measurement of the permeability of individual glomeruli in a fast a reproducible manner. An increase in the glomerular L_p_A/V_i_ indicates disruption of the GFB, which is suggestive of renal disease.

1. Set up the glomerular L_p_A/V_i _rig as described in Salmon *et al*^10^. Please refer to Figure 1 for a detailed diagram of the set up.
2. Prepare the following solutions: mammalian Ringer's solution with 1% BSA (pH 7.4) and mammalian Ringer's solution with 8% BSA (pH 7.4). Warm both to 37 °C.
3. Pull micropipettes from glass capillary tubes (optical density: 1.2 mm). Generate a 5 - 8 µm aperture tip by cutting the micropipette under a microscope.
4. Use the glomerular L_p_A/V_i _rig to catch intact individual glomeruli that are free of Bowman's capsule and tubular fragments onto the micropipette using suction. A detailed summary of the oncometric assay is found in Salmon *et al*^10^. In brief, once a glomerulus is caught and secured on the suction micropipette, begin recording the video of the glomerulus under the microscope.
5. Firstly, equilibrate the glomerulus in the 1% BSA Ringer's solution for 30 s before switching the perifusate to the concentrated 8% BSA Ringer's solution for 10 s. Then switch the perifusate back to 1% BSA Ringer's solution and stop the recording.
6. Wash the glomerulus away and repeat the process for 10 - 15 glomeruli per mouse. Ensure the perifusate flow rates are identical and not to fast (10 mL/min) so as not to distort the glomerular structure.
7. Measure the initial rate of glomerular shrinkage to calculate the glomerular water permeability (L_p_A) normalized to the glomerular volume (V_i_). Detailed information regarding the analysis can be found in Salmon *et al*^10^.

### 6. Periodic Acid Schiff (PAS) Stain

NOTE: The PAS stain will highlight the basement membranes of glomerular capillary loops and the tubular epithelium. It enables detailed visualization of the glomerular cells, mesangial matrix and potential expansion, and potential changes of the GBM (*i.e.*, thickening and irregularities).

1. Section the paraffin-embedded, PFA-fixed kidney cortex using a microtome at 5 µm thickness onto poly-L-lysine coated slides. Dry at 37 °C for 1 h. Ensure the section does not contain any folds or holes, which can distort the morphology under the microscope.
2. Deparaffinize slides by incubating twice in xylene (CAUTION, irritant; use in fume cabinet) for 3 min each, twice in 100% EtOH for 3 min each, and then once in 95%, 70%, and 50% EtOH for 3 min each, all at room temperature. Re-hydrate the slides in dH_2_O.
3. Incubate the slides in periodic acid solution (CAUTION, irritant; use in fume cabinet) (1 g/dL) for 5 min, and then rinse the slides in several changes of dH_2_O. Use a container with 100 mL dH_2_O at room temperature. CAUTION: periodic acid solution: irritant; use in fume cabinet
4. Incubate slides in Schiff's reagent (Parasoaniline HCl 6 g/L and sodium metabisulfite 4% in HCl 0.25 mol/L) for 15 min at room temperature. Wash slides in running tap water for 5 min.
5. Counterstain with Hematoxylin for 3 s before thoroughly rinsing slides in running tap water for 15 min. NOTE: Some optimization may be required to determine the optimal time for Hematoxylin staining.
6. Dehydrate slides using the reverse of the deparaffinization protocol in step 6.2. Finish with xylene.
7. Air dry slides and mount with xylene-based mounting media.
8. Image on a light microscope at 400X magnification to assess glomerular structures. Evaluate the following: thickening and irregularities of the GBM, collapsing of capillary loops, fibrotic tissue, sclerosis, cellular proliferation (endothelial, podocyte, and mesangial, or inflammatory cells infiltrating the tuft). NOTE: For a comprehensive evaluation of glomerular pathophysiology, lesions elsewhere in the kidney should be evaluated, such as in the tubules.

### 7. Transmission Electron Microscopy (TEM)

NOTE: TEM allows the examination of ultra-structural abnormalities in the kidney, such as the GBM, podocyte foot processes, and endothelial fenestrations, which are not visible with light microscopy. This is important in models where renal damage is not so pronounced (*i.e.*, no albuminuria and major structural abnormalities).

1. Take the 2.5% gluteraldehdye- fixed diced kidney and post-fix in 1% osmium tetroxide for 1 h. Wash in 50 mL of 0.1 M cacodylate buffer (pH 7.3) and then 50 mL of dH_2_O (3 x 15 min changes). CAUTION: 2.5% glutaraldehyde: toxic, sensitizer, irritant; use in a fume cabinet. 0.1 M sodium cacodylate: toxic, use in a fume cabinet
2. Dehydrate with EtOH and embed in Araldite resin.
3. Cut sections at 50 - 100 nm thickness and stain with 3% (aqueous) uranyl acetate and Reynolds' lead citrate solution.
4. Take digital micrographs over several areas of the glomerulus at 940X, 1250X, and 6200X to be sure the podocytes, GEnCs, GBM, and mesangium can be identified.
5. Use ImageJ to analyze the blinded glomeruli. Set the scale for each 6200X micrograph by drawing a line between two points of known distance, such as a ruler. Go to analyze, and then set scale, where the length of the line will be displayed in pixels. Type in the known distance and units of measure. Use the protocols listed below for the measurement of each parameter. NOTE: This analysis requires around 1 day per mouse. For adequate statistical power, use the average measurements from 3 glomeruli from 3 mice.
  1. For GBM, insert a fixed digital grid (10 x 10) over the 6200X micrograph and measure the thickness of the GBM at the point where the grid lines cross the GBM. Measure from the basal endothelial cell membrane to the basal podocyte foot process cell membrane in a perpendicular tangent to the endothelial cell membrane using the straight line tool. Determine the mean measurement for each glomerulus from 10 individual measurements.
  2. For the endothelial fenestration number, measure the length of the GBM present in the 6200X micrograph and count the number of endothelial fenestrations per unit length of GBM. Take an average from at least 4 micrographs per glomerulus.
  3. For the podocyte foot process width, insert a fixed digital grid (10 x 10) over the 6200X micrograph. Measure the width of the podocyte foot processes that cross the grid lines. Measure the width at the widest part of the foot process where it meets the GBM; ensure the line is perpendicular to the tangent of the podocyte basal membrane. Determine the mean measurement for each glomerulus from 10 individual measurements.
  4. For podocyte slit width, insert a fixed digital grid (10 x 10) over the 6200X micrograph. Measure the width of the podocyte slit diaphragms that cross the grid lines. This is the point where the foot processes are closest together, at the widest part of each foot process, from podocyte membrane to membrane. Ensure the measurement is perpendicular to the tangent of the podocyte basal membrane. Determine the mean measurement for each glomerulus from 10 individual measurements.
  5. For the number of podocyte foot processes, measure the length of the GBM present in the 6200X micrograph and count the number of podocyte foot processes per unit length of GBM. Take an average from at least 4 micrographs per glomerulus.
  6. For the sub-podocyte space coverage, see detailed method in Neal *et al*.^14^.
6. Using the 940X micrographs, examine glomeruli for the presence of abnormal structure, deposits, and infiltrates by eye.

### 8. Immunofluorescence for Podocyte and Endothelial Markers

NOTE: Immunostaining allows visualization of the protein expression patterns, such as endothelial capillary loops, which can collapse in glomerular disease.

1. Place the OCT-mold containing frozen kidney at -20 °C for 2 h prior to sectioning. Ensure the cut surface of the kidney is carefully placed against the bottom of the OCT-mold to enable well orientated tissue sections.
2. Using a cryostat, section tissue at a 5 µm thickness on to poly-L-lysine coated slides. NOTE: Ensure there are no folds or holes in the tissue section, which can distort the morphology.
3. Upon removal from the cryostat, fix slides in 4% PFA for 10 min. Wash slides 3 x 5 min in a container with 100 mL of dH_2_O. CAUTION: 4% PFA: fixative, use in fume cabinet
4. To minimize the amount of antibody used, draw around sections with a hydrophobic pen. Do not let the sections dry.
5. Incubate in blocking solution (3% BSA and 5% normal serum in 1x PBS) for 1 h at room temperature.
6. Remove the blocking solution with an aspirator and incubate sections with primary antibody (Nephrin, podocin, or PECAM-1) diluted 1:250 (3% BSA in 1x PBS). Place slides in a humidified chamber at 4 °C overnight. If a humid chamber is not available, lightly place a small strip of parafilm over the antibody-coated slide. Take care when removing the next day as to not disrupt the section.
7. Wash slides 3 x 5 min in 1x PBS.
8. Incubate with appropriate fluorescent secondary antibody dilution 1:1000 (3% BSA in 1x PBS) for 2 h at room temperature in the dark.
9. Wash slides 3 x 5 min in 1x PBS. Mount with fluorescent mounting media containing DAPI.
10. Image slides with a fluorescent microscope at 400X magnification to view the glomeruli. Ensure this is blinded to avoid bias.
11. Use ImageJ to analyze the staining intensity, normalized to the glomerular area, and the pattern of staining, *i.e.*, number of capillary loops normalized to the glomerular area, in a blinded manner.

### 9. Protein Extraction and Western Blotting

NOTE: Western blotting allows us to assess the expression proteins known to be dysregulated in renal disease. For example, a reduction in podocin and nephrin expression indicates podocyte loss.

1. Extract protein from kidney cortex and sieved glomeruli; the protocol is the same for each and the volume of lysis buffer is adjusted for the amount of tissue.
2. Thaw kidney/glomeruli on ice before adding NP-40 lysis buffer (150 mM NaCl, 1% NP-40, 50 mM Tris pH 8) containing protease and phosphatase inhibitors. Homogenize the sample for 30 s.
3. Incubate the homogenized samples on ice for 30 min, vortexing at regular intervals.
4. Centrifuge the samples at 10,000 x g for 15 min at 4 °C.
5. Remove the supernatant to a fresh tube on ice. NOTE: expect to recover approximately 1 mg protein per sample.
6. Denature the proteins using the standard 4x Laemmli buffer. Boil the mixture at 95 - 100 °C for 5 min.
7. Assess the expression of glomerular cell marker proteins (Nephrin, Podocin, PECAM-1, *etc*.) and the phosphorylation and expression of proteins known/hypothesized to be altered in the kidney/glomeruli of the disease model using Western blotting (standard method; Mahmood and Yang^15^). NOTE: The protocol will vary depending on the size and abundance of the protein of interest.

### 10. RNA Extraction and Polymerase Chain Reaction (PCR)

NOTE: mRNA expression analysis allows us to determine how genes are regulated in renal disease, such as changes in gene expression and alternative splicing.

1. Whilst the kidney cortex is still frozen, thoroughly grind in 3 mL of phenol reagent using a pestle and mortar. If using glomerular extracts, add 1 mL of phenol reagent and homogenize the sample for 30 s. CAUTION: TRIzol reagent: irritant; use in fume cabinet
2. Perform an RNA extraction using the method described by Chomczynski and Sacchi^16^. NOTE: Commercial RNA extraction kits are available as an alternative to this method.
3. Assess the quantity and quality of RNA obtained using one of the various methods available. RNA is aliquoted and stored at -80 °C at this point. Avoid repeat freeze thawing. NOTE: If new to this method, check the quality of the RNA before proceeding to the next step by running the RNA on an agarose gel to ensure a clear 28S and 18S ribosomal band. Expect to recover between 2 to 5 µg of RNA using this method.
4. DNase treat 1 µg of RNA (make volume up to 10 µL with RNase-free water plus 1 µL of DNase and 1 µL of DNase buffer) for 1 h at 37 °C. Stop the reaction with 1 µL of DNase stop solution at 65 °C for 10 min.
5. Add 0.5 µL of oligo (dT) and random primers. Incubate at 70 °C for 10 min.
6. Immediately quench on ice for 5 min.
7. Add the following; MMLV reverse transcriptase enzyme (400 U; replace with DEPC H_2_O in the RT - control sample), MMLV buffer (1x), dNTP mix (0.5 mM), and ribonuclease inhibitor (40 U); make up to 50 µL with DEPC water.
8. Incubate reaction mix at 37 °C for 1 h followed by 95 °C for 5 min to deactivate the enzyme. NOTE: To generate a higher yield of cDNA, incubate at 37 °C for up to 3 h.
9. Assess the quantity and quality of cDNA using the various methods available.
10. Use PCR to assess the mRNA expression and splicing patterns of genes hypothesized to be dysregulated in the glomerular disease model. The protocol will vary depending on the gene of interest.

## Representative Results

Urine was collected using metabolic cages from wild type (WT), inducible podocyte-specific VEGF-A knock out (VEGF-A KO), and VEGF-A KO X Neph-VEGF_165_b mice (VEGF-A KO mice that over-express the human VEGF-A_165_b isoform in the podocytes in a constitutive manner). Upon measurement of the urinary albumin creatinine ratio at weeks 0, 4, 10, and 14 after doxycycline induction of VEGF-A KO, VEGF-A KO mice developed progressive albuminuria by 10 weeks compared to WT littermate controls. The absolute values can be seen in Figure 2A, and the normalized to the baseline of each mouse values in Figure 2B. However, albuminuria in not observed in the VEGF-A X Neph-VEGF-A_165_b mice (Figure 2), indicating that VEGF-A_165_b is protective in the model of albuminuria^5^.

The glomerular L_p_AV_i_ was measured in individual glomeruli sieved from WT, VEGF-A KO, and VEGF-A X Neph-VEGF-A_165_b kidneys. An example of how a glomerulus is caught and the shrinkage observed when perifused with 8% BSA is shown in Figure 3A. This shrinkage is then used to determine the glomerular L_p_A/V_i_ for each glomerulus (Figure 3B). VEGF-A KO mice had a significantly increased glomerular L_p_A/V_i_ at 14 weeks post VEGF-KO induction compared to WT control glomeruli. Although lower in VEGF-A X Neph-VEGF-A_165_b mice, the increased glomerular L_p_A/V_i_ was not prevented by over-expression of VEGF-A_165_b at 14 weeks^5^.

PAS staining of kidney cortex sections 14 weeks after induction of VEGF-A KO did not reveal any glomerular structural abnormalities in the VEGF-A KO or VEGF-A X Neph-VEGF-A_165_b mice (Figure 4A). However, upon analysis of the glomerular ultra-structure via EM, VEGF-A KO mice had developed an increased GBM width, decreased number of endothelial fenestrae, decreased SPS coverage, and increased average podocyte slit width (Figure 4C**-4F**). The average podocyte foot process width and number of slits remained unchanged (Figure 3B** and 3G**). Over-expression of VEGF-A_165_b in the VEGF-A KO mice prevented the changes to the GBM and slit width (Figure 4C** and 4F**). However, VEGF-A_165_b had no effect on the altered fenestrae number and SPS coverage (Figure 4D** and 4E**)^5^.

RT-PCR performed on RNA extracted from sieved glomeruli revealed that the human VEGF-A_165_b mRNA is only present in the VEGF-A KO X Neph-VEGF-A_165_b mice (Figure 5A). When extracting protein from sieved glomeruli and assessing the levels of proteins via Western blotting, the glomerular protein expression of VEGFR-2 was found to be decreased in VEGF-A KO mice, which was prevented by over-expression of VEGF-A_165_b (Figure 5B** and 5C**)^5^.

**Figure:**
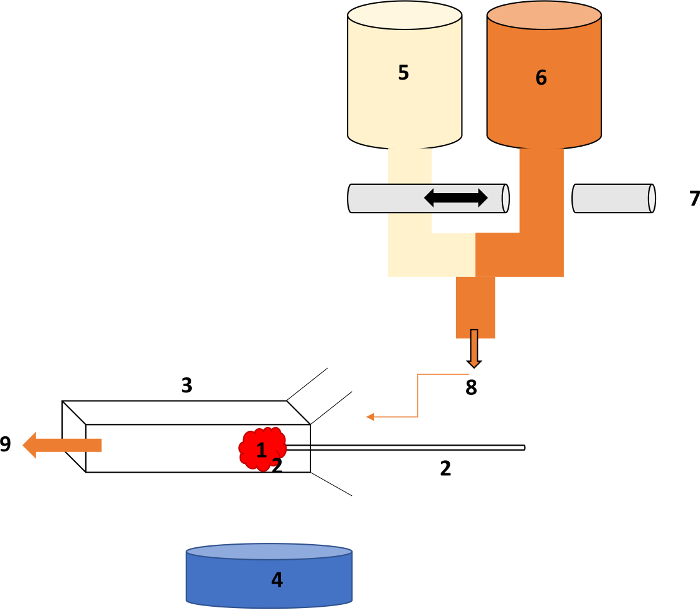

**Figure 1. Diagrammatic set up of the glomerular permeability (L_p_A) rig.(A)** The glomerulus is caught on **(B)** the micropipette within a holder using suction, which is clamped onto a mount for stability. **(C) **Rectangular microslide. **(D)** 4X objective of a microscope with a video camera. **(E)** 1% BSA solution warmed to 37 °C. **(F)** 8% BSA solution warmed to 37 °C. **(G) **Remote tap bearing two perifusate-containing lines, which permits rapid perifusate exchange. **(H) **Route of perifusate towards the microslide, which then bathes the glomerulus. Please click here to view a larger version of this figure.

**Figure:**
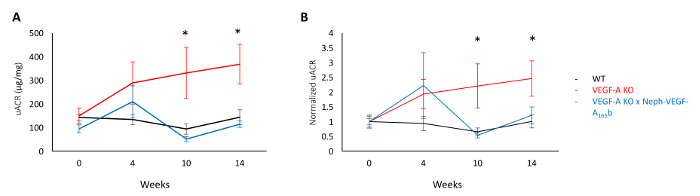

**Figure 2. Urinary albumin creatinine ratio. (A)** The uACR values at weeks 0, 4, 10, and 14 after induction of VEGF-A KO in WT, VEGF-A KO, and VEGF-A X Neph-VEGF-A_165_b mice. **(B)** The same uACR values normalized to the baseline value (week 0) of each individual mouse. The uACR significantly increased in VEGF-A KO mice at weeks 10 and 14 compared to WT littermate controls, which was prevented in the VEGF-A X Neph-VEGF-A_165_b mice (*p <0.05; Two-way ANOVA, correction for comparison between pairs; n= 4 - 12 mice per time point; error bars: standard error of the mean [SEM]). This figure has been modified from Stevens *et al*^5^. Please click here to view a larger version of this figure.

**Figure:**
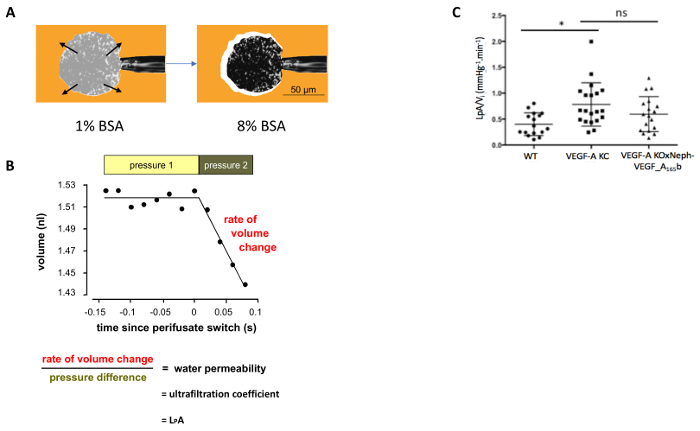

**Figure 3. Measurement of glomerular water permeability. (A)** The glomerulus is caught on the micropipette via suction; the perifusate is switched from 1% BSA (Ai) to 8% BSA (Aii), and glomerular shrinkage is observed. **(B)** Measurements taken before and after the 8% BSA switch are used to determine the glomerular L_p_A/V_i_. **(C)** VEGF-A KO mice develop an increased glomerular L_p_A/V_i_ at 14 weeks post induction of VEGF-A KO compared to WT controls. This was not significantly prevented in VEGF-A X Neph-VEGF-A_165_b mice (*p <0.05; One way ANOVA, Bonferroni correction for comparison between pairs; n = 4 - 9 mice, 15 - 30 glomeruli; error bars: SEM). Please click here to view a larger version of this figure.

**Figure:**
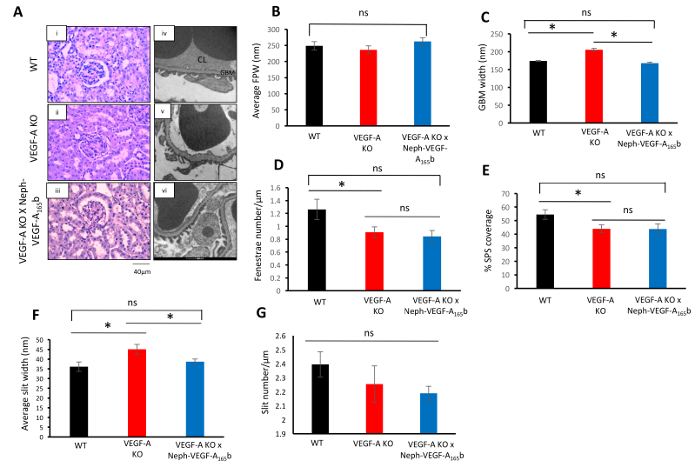

**Figure 4. Glomerular structural analysis. (A)** PAS staining of the kidney cortex did not indicate any structural abnormalities in the glomeruli from WT, VEGF-A KO, and VEGF-A X Neph-VEGF-A_165_b mice (Ai - iii). EM revealed ultra-structural abnormalities in the VEGF-A KO glomeruli (Aiv - vi). **(B)** The average FPW did not change between groups. **(C)** The GBM increased in the VEGF-A KO glomeruli, which was prevented by VEGF-A_165_b. **(D)** The number of fenestrae was decreased in VEGF-A KO glomeruli, which remained unaltered by VEGF-A_165_b. **(E)** The SPS coverage was decreased in VEGF-A KO glomeruli, which also remained unchanged by VEGF-A_165_b. **(F) **The average slit width was increased in VEGF-A KO glomeruli, which was prevented by VEGF-A_165_b. **(G)** The slit number was unchanged between the three groups (*p <0.05; One way ANOVA, Bonferroni correction for comparison between pairs; n = 3 mice, 9 glomeruli; error bars: SEM). This figure has been modified from Stevens *et al*^5^. Please click here to view a larger version of this figure.

**Figure:**
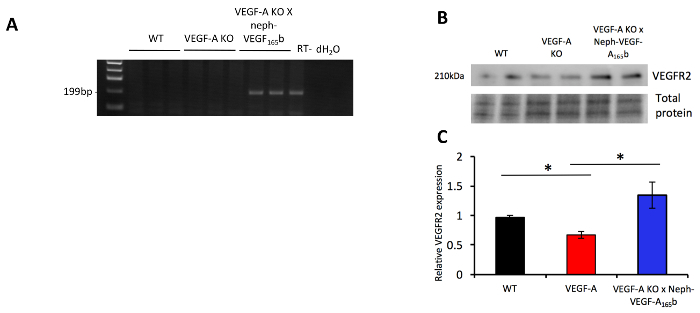

**Figure 5. mRNA and protein expression of markers. (A)** RT-PCR showed that human VEGF-A_165_b mRNA expression was only evident in the sieved glomeruli from VEGF-A KO X Neph-VEGF-A_165_b mice. **(B)** Western blotting indicated that VEGFR-2 protein expression was down-regulated in VEGF-A KO glomeruli, which was prevented in VEGF-A KO X Neph-VEGF-A_165_b glomeruli (*p <0.05; One way ANOVA, Bonferroni correction for comparison between pairs; n = 3-  6 mice; error bars: SEM). This figure has been modified from Stevens *et al*^5^. Please click here to view a larger version of this figure.

## Discussion

This protocol describes a full kidney work-up that should be carried out in mouse models of glomerular disease, enabling a vast amount of information regarding kidney and glomerular function to be obtained from a single mouse. The critical steps in each method allow for detailed functional, structural, and mechanistic analysis of glomerular function, including assessment of the permeability of the kidneys as a whole (uACR and plasma creatinine measurements), the permeability of individual glomeruli (glomerular L_p_A/V_i_), examination of the structural alterations (PAS, Trichrome blue, and EM), protein localization (IF), and glomerular gene expression (RT-PCR and Western blotting). These methods are key to the full assessment of glomerular function in mouse models of renal disease.

When assessing the permeability of the GFB, many studies have opted to use the conventional uACR or 24 h albumin excretion rate as an effective measure^17^,^18^. Although these techniques allow assessment of the GFB permeability as a whole, it does not allow for individual glomerular permeability assessment and variation amongst glomeruli. Previous studies have found measurement of the glomerular L_p_A/V_i_ to be a more sensitive measure of changes to the GFB permeability^5^,^9^. Indeed, in the representative results demonstrated in this paper, at 14 weeks post induction of VEGF-A KO, VEGF-A KO X Neph-VEGF-A_165_b mice have a significantly lower uACR compared to VEGF-A KO mice; however, this result is not reflected in the glomerular L_p_A/V_i_ measurements, where VEGF-A_165_b did not significantly prevent increases in the GFB permeability (Figure 1 and Figure 2)^5^. This shows the importance of using multiple assays to assess both the kidney permeability and the permeability of individual glomeruli. Furthermore, the glomerular L_p_A/V_i_ oncometric assay suggests that the permeability of individual glomeruli from the same kidney can vary greatly, especially in disease models^5^,^10^,^19^. One limitation to measuring the glomerular L_p_A/V_i_ is that it can only be performed at the experimental end-point; thus, regular uACR measurements are required to give an indication of the experimental end-point.

In addition to assessing the functional phenotype, the present method also encourages assessment of the structural and ultrastructural phenotype. This can be done using a selection of stains such as PAS, trichrome, and silver stains; each to assess different aspects of the glomerular morphology. In acute models of glomerular disease, which is often the case in mouse models, is can be difficult to detect any major structural abnormalities using these stains unless you are a trained renal pathologist. Therefore, carrying out EM is suggested to assess the ultrastructure of the GFB, which allows the quantitative measurement of parameters such as the GBM, endothelial fenestrae size and number, and podocyte characteristics. Such measurements require minimal training to perform and enables the investigator to determine the cell-types/structures affected in a disease model. In the example shown in the representative results, the VEGF-A KO mouse was found to be a mild model of glomerular disease, thus, no major structural abnormalities were present upon PAS staining. However, podocyte-specific VEGF-A KO did induce changes to the GBM, podocytes, and endothelial cells when examining the glomerular ultra-structure^5^. Unfortunately, the preparation of the kidney for EM described in the present method does not enable detection of the endothelial glycocalyx, which is also known to have significant effects on the permeability of the GFB^19^. In order to accurately measure the glycocalyx depth, the kidney should be perfuse-fixed with 2.5% glutaraldehyde with 1% Alcian blue for endothelial glycocalyx labelling, as described in Oltean *et al*^19^.

Once the functional and structural phenotype have been assessed, the expression/activation patterns of different genes and pathways can then be assessed specifically in the glomeruli. Prior ultra-structural assessment could give some information regarding the cell types/glomerular structures involved, indicating whether podocyte- or endothelial-specific genes/pathways should be examined. For example, in the representative results from the VEGF-A KO mice, a reduction in the endothelial fenestrae number was observed (Figure 3D); therefore, the glomerular protein expression of an endothelial marker known to be involved in the VEGF-A pathway was examined; VEGFR-2 (Figure 4B)^5^. In addition to the expression of proteins in the glomeruli, their localization can also be visualized using IF. In a study by Zhang *et al*^20^, podocyte-specific overexpression of GLUT1 was confirmed in the podocytes by IF co-localizing the increased GLUT1 with podocin.

In comparison to alternative methods presented in the literature to assess glomerular function, the use of the method outlined in this paper to assess kidney function in mouse models of glomerular disease enables the glomerular phenotype to be fully evaluated from multiple aspects. By using this method, the researcher is able to determine the kidney phenotype of the model and assess the mechanism as to why the phenotype develops. This vital information on the mechanism of disease is required when examining potential therapeutic avenues in these models. This method can be easily applied to future investigations into glomerular function both in the assessment of disease phenotypes and potential therapeutics.

In conclusion, this generic and adaptable protocol describes a full kidney work-up for mouse models of glomerular disease, enabling a vast amount of information regarding kidney and glomerular function to be obtained from a single mouse. The methods allow for detailed functional, structural, and mechanistic analysis of glomerular function, which can be applied to all mouse models of glomerular disease.

## Disclosures

The authors have nothing to disclose.
